# Biochanin A restored the blood–brain barrier in cerebral ischemia-reperfusion in rats

**DOI:** 10.1590/1806-9282.20240025

**Published:** 2024-07-19

**Authors:** Abdulmutalip Karaaslanli, Fırat Aşir, Görkem Tutal Gürsoy, Mehmet Cudi Tuncer

**Affiliations:** 1 Ergani State Hospital, Department of Brain and Neurosurgery – Diyarbakır, Turkey.; 2 Dicle University, Faculty of Medicine, Department of Histology and Embryology – Diyarbkır, Turkey.; 3 Ankara City Hospital, Department of Neurology – Ankara, Turkey.; 4 Dicle University, Faculty of Medicine, Department of Anatomy – Diyarbakir, Turkey.

**Keywords:** Blood–brain barrier, Artery occlusion, Antioxidant, Cerebrum, Endothelium

## Abstract

**OBJECTIVE:**

Blood–brain barrier is a protective layer that regulates the influx and efflux of biological materials for cerebral tissue. The aim of this study was to investigate the effects of Biochanin A on cerebral histopathology and blood–brain barrier immunohistochemically.

**METHODS:**

A total of 24 rats were assigned to three groups: sham, ischemia-reperfusion, and ischemia-reperfusion+Biochanin A. Ischemia-reperfusion was performed by occluding the left carotid artery for 2/24 h. Notably, 20 mg/kg Biochanin A was administered to rats for 7 days after ischemia-reperfusion. Blood was collected for malondialdehyde and total oxidant/antioxidant status analysis. Cerebral tissues were processed for histopathology and further for immunohistochemical analysis.

**RESULTS:**

Malondialdehyde content with total oxidant status value was significantly increased and total antioxidant status values were significantly decreased in the ischemia-reperfusion group compared with the sham group. Biochanin A treatment significantly improved scores in the ischemia-reperfusion+Biochanin A group. The normal histological appearance was recorded in the cerebral sections of the sham group. Degenerated neurons and vascular structures with disrupted integrity of the cerebral cortex were observed after ischemia-reperfusion. Biochanin A alleviated the histopathology in the cerebrum in the ischemia-reperfusion+Biochanin A group. Ischemia-reperfusion injury decreased the expression of blood–brain barrier in the ischemia-reperfusion group compared to the sham group. Administration of Biochanin A upregulated the blood–brain barrier immunoreactivity in the cerebrum by restoring blood–brain barrier.

**CONCLUSION:**

Cerebral ischemia-reperfusion caused an increase in oxidative stress and pathological lesions in the cerebrum. Biochanin A treatment restored the adverse effects of ischemia-reperfusion injury by restoring blood–brain barrier.

## INTRODUCTION

Brain is an element of the central nervous system that contains numerous nerve cells. Homeostasis and function of the brain are important to elucidate brain damage^1,2^. The cross-ancestry genetic risk score has been reported to predict ischemic stroke independently of clinical risk factors and outperform previous genetic risk assessment^3^. It has been stated that Biochanin A (BCA) shows protective effects in angiotensin II-induced model rats and may cause an increase in endophilin A2 expression and a decrease in angiotensin II type 1 receptor expression due to the inhibition of inflammatory responses^4^. BCA (C16H12O5) is an O-methylated natural flavonoid found in red clover, chickpeas, and other legumes, belonging to the phytoestrogen family^5^. Recent studies showed that BCA has various pharmacological properties, including anti-tumorigenesis, anti-oxidation, anti-inflammatory, and hypoglycemic effects^6-8^. BCA was reported to be effective in the treatment of cerebral Ischemia-reperfusion (IR) injury in rats^9,10^. BCA was also specifically shown to prevent the initiation of inflammatory response and downregulate the expression of pro-inflammatory factors in rats^9,11^.

Cerebral IR can clinically cause vasogenic edema and hemorrhagic transformation and may result in mortality if not treated in the acute phase^12^. SMI71 is a specific marker to show rat blood–brain barrier (BBB). Many studies showed that SMI71 could be used to investigate BBB integrity^13^. SMI 71 is an antibody designed for detecting a rat endothelial protein localized in regions containing BBB. SMI71 does not react with endothelial cells in periventricular and peripheral tissues such as the liver, heart, adrenal glands, skeletal muscle, intestine, thymus, lymph nodes, pancreas, thyroid, and skin. Notably, the reactivity with this antibody emerges in newborn rats concurrently with the maturation of the BBB^14,15^.

This study aimed to investigate the effectiveness of BCA on the histology of BBB after cerebral IR by examining the expression level of components of the BBB.

## METHODS

### Ethical approval and animal housing

All animal experiments were approved by the Animal Experimentation Local Ethics Committee of Dicle University (2023/04). Animals were allowed access to water and food *ad libitum* and housed in cages (12 h/12 h dark/light period, 23±1°C). BCA was purchased from Merck (catalog no: D2016, Germany).

### Surgical procedures

All procedures were performed under anesthesia. A total of 24 Wistar albino female rats were assigned to three categories: sham, IR, and IR+ BCA (n=8 per group). The rats were fixed on the operating table in the supine position, and the neckline was cleaned with povidone iodine. Using surgical scissors, a midline incision was made from the upper edge of the sternum to the hyoid bone. The incision area was enlarged using a tissue retractor through the trachea. Then, the paratracheal muscles were dissected, and the common carotid artery (CCA) was observed. CCA was occluded for 2 h via a micro bulldog clamp on the left CCA approximately 1 cm proximal to the carotid bifurcation. After cerebral Ischemia, the clamp was removed, the tissues were placed back to their anatomical location, and the skin and subcutaneous fascia were sutured. Cerebral reperfusion was allowed for 24 h. A 200 mM stock solution was prepared by dissolving BCA in DMSO solution.

1. **Sham group:** Cerebral artery occlusion was not performed. Only the left CCA was isolated and placed back to anatomical location. Animals were given 1 mL of DMSO intraperitoneally for 7 days.
2. **IR group:** Cerebral IR procedure was performed. Animals were given 1 mL of DMSO intraperitoneally for 7 days.
3. **IR+BCA group:** After IR treatment, 20 mg/kg BCA was administered to rats intraperitoneally for 7 days.

### Malondialdehyde and total antioxidant status/total oxidant status

At the end of the experimental protocol (at the end of the seventh day), all animals were sacrificed under anesthesia. Malondialdehyde (MDA, MERCK, catalog no: MAK085), total antioxidant (TAS, mmol Trolox Equiv./L), and oxidant status (TOS, μmol H_2_O_2_ Equiv./L) kits were commercially purchased (Rel Assay Diagnostics, Turkey). Blood samples of each rat were centrifuged at 2000 rpm for 10 min, and the supernatant was collected. The measurement of MDA, TAS, and TOS was done. Serum plasma of blood samples were further analyzed for MDA, TAS, and TOS levels that were determined according to Durgun et al.^16^

### Histological tissue processing

Cerebral tissues were excised for histological sampling. Dissected cerebral samples were further analyzed for histological evaluation. Samples were immersed in zinc-formalin, dehydrated through grading alcohol series, and incubated in paraffin wax. Sections of 5 μm were cut from paraffin blocks and stained for hematoxylin–eosin dye and immunostaining^17^.

### Immunohistochemical examination

Cerebral sections were dewaxed, hydrated in grading alcohol series, and washed in distilled water. Hydrogen peroxide (H_2_O_2_; 3%) was dropped on slides to block endogen peroxidase activity. After washing in PBS, sections were incubated with anti-BBB (catalog no: 836804, Biolegend, California, USA), overnight at+4°C. Sections were biotinylated and allowed to react with streptavidin peroxidase solution (Thermo Fischer, USA) for 15 min. After PBS washing, diaminobenzidine (DAB) chromogen was used as a chromogen to observe color change. The reactions were stopped with PBS solution, and sections were counter-stained with hematoxylin dye. Slides were mounted and imaged with Zeiss Imager A2 light microscope. All images were processed and quantified using the ImageJ software. Negative control staining was done similar to the same protocol, but only sections were incubated with PBS instead of an antibody of interest.

### Image J analysis

The staining intensity of BBB expression was measured by the Image J software (version 1.53, http://imagej.nih.gov/ij). Measurement was performed by the method of Crowe et al.^18^. Quantification was recorded by analyzing 10 fields from each specimen per group^19^. In specimens, the brown color stands for the positive expression of the antibody of interest, while the blue color represents a negative expression of the antibody of interest. Signal intensity (expression) from a field was calculated by dividing the intensity of the antibody of interest by the whole area of the specimen. A value for staining area/whole area was calculated for each specimen from ten fields. An average value was measured for groups and analyzed for semi-quantitative immunohistochemistry scoring.

### Statistical analysis

Statistical analysis was done using the IBM SPSS 25.0 software (IBM, Armonk, New York, USA). Data distribution was done by the Shapiro-Wilk test. The data were recorded as median (IQR). The non-parametric Kruskal-Wallis test was used for analyses between more than two groups, and the post-hoc Dunn test was used due to the small number of animals in the groups. Statistical significance was accepted for values p<0.05.

## RESULTS

### Oxidative stress findings

Statistical analysis of biochemical and histopathologic scores is shown in Table 1. MDA and TOS values were significantly increased in the IR group compared with the sham group. TAS value was statistically decreased in the IR group compared with the sham group. After BCA treatment, MDA and TOS levels statistically decreased and TAS content statistically increased in the IR+BCA group compared with the IR group.

**Table 1:** Evaluation of biochemical parameters and Image J analysis of blood–brain barrier signal in groups.

Groups	MDA	TAS	TOS
Sham	1.05 (0.93–1.19)	4.47 (4.20–6.33)	4.63 (3.94–6.02)
IR	7.18 (6.11–7.95)	1.23 (1.12–1.31)	16.71 (15.25–18.37)
IR+BCA	3.33 (2.33–4.43)	1.42 (1.38–1.50)	8.57 (7.43–9.66)
Dunn's test	<0.01*	<0.01*	<0.01*
	0.022**	0.024**	0.022**
Groups	BBB signal	Kruskal-Wallis	Dunn's test
Sham	39.41 (35.20–42.98)	0.025	0.028*
IR	32.91 (26.39–35.33)		0.048**
IR+BCA	37.86 (34.21–40.68)		

Data were presented as median (IQR),

* sham vs. IR,

** IR vs. IR+BCA.

### Histopathologic findings

**Figure 1:**
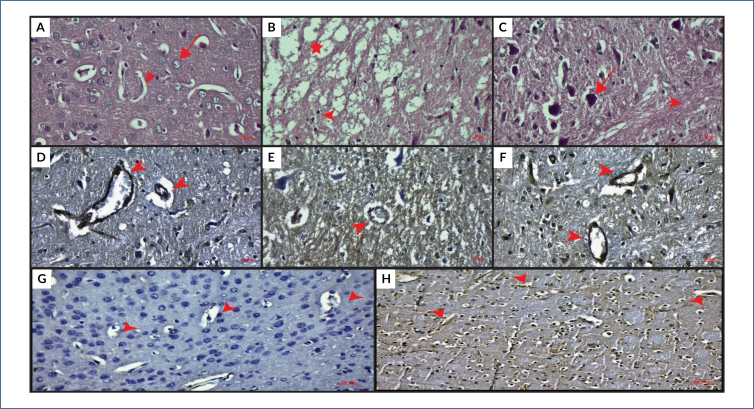
Hematoxylin–eosin staining of the cerebrum. (A) Histologically normal neurons (arrow) and capillaries (arrowhead) in the sham group. (B) Cerebral pathologies in the IR group. Disrupted cortex integrity (star) and pyknotic neurons (arrow). (C) Histologically normal neurons (arrow) and axon fibers (arrowhead). Immunoreactivity of blood–brain barrier in cerebral section (D) Sham group, (E) Ischemia-reperfusion group, and (F) Ischemia-reperfusion+Biochanin A group. Arrowheads: capillaries. (G) Negative control. (H) Positive control of the cerebral section of the healthy rat. Scale bar: 20 μm; Original magnification: 40×.

Hematoxylin–eosin staining of cerebral sections is shown in Figures 1A–C. The sham group showed no pathological lesions in the cerebrum. Neurons were histologically normal along with normal vessels (Figure 1A). In the IR group, cerebral cortex integrity was disrupted with degenerated neurons and vascular structures. A high number of cells were with pyknotic nucleus (Figure 1B). Compared with the IR group, BCA treatment restored the cerebral pathologies after IR in the IR+BCA group (Figure 1C). BBB immunoreactivity is shown in Figures 1D–F. High expression of BBB was recorded in the sham group around the blood vessels where the nerve–blood barrier existed (Figure 1D). BBB immunoreactivity was decreased in IR due to the disruption of BBB (Figure 1E). Post-BCA treatment increased the BBB immune activity by restoring the BBB in the cerebral cortex. BBB immune reactivity was intensely observed around the regions where the barrier existed compared with the IR group (Figure 1F). Negative and positive control immunostaining of the cerebral section of the healthy rat is shown in Figures 1G and H, respectively.

### Image J analysis

**Figure 2:**
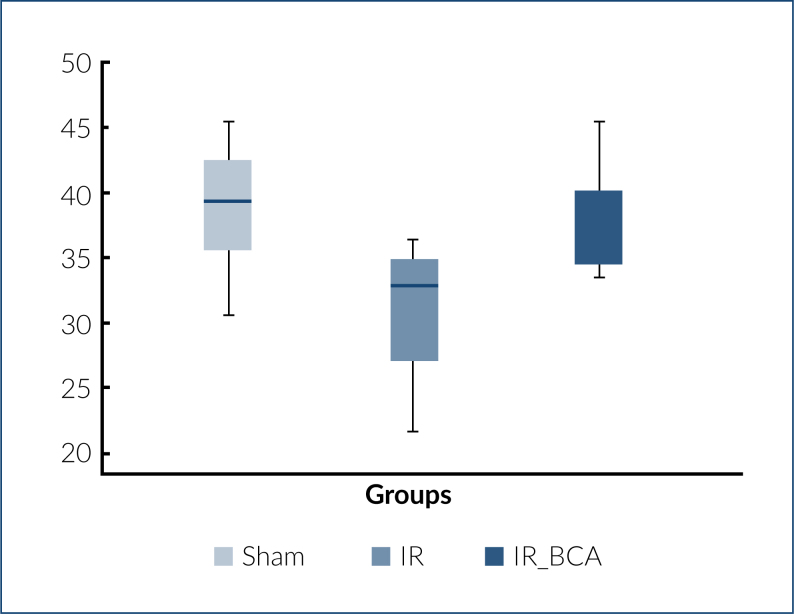
Box plot of blood–brain barrier signal (expression) per group.

The staining intensity of BBB expression is shown in Figure 2. BBB expression was downregulated after cerebral IR injury. However, BCA treatment upregulated BBB expression with its antioxidant properties.

## DISCUSSION

IR injury is listed as one of the causes of tissue damage in clinics such as myocardial infarction, stroke, and organ transplantation. After re-blooding of tissue, more damage occurs in IR as the paradox of IR injury. This process is quite complex and not fully understood yet^20^. During IR injury, the production of reactive oxygen species (ROS) increases, and alterations in mitochondrial homeostasis lead to oxidative damage in tissues and eventually induce the proinflammatory response^21,22^. Medicinal plants with antioxidant activity alleviate the IR injury^23-25^. BCA is a medicinal plant that has a similar action to melatonin^26^. BCA exhibits antioxidant properties, which can help neutralize ROS generated during reperfusion. By scavenging ROS, BCA may reduce oxidative stress and prevent cellular damage^27^. Additionally, IR injury triggers an inflammatory response, leading to tissue damage. BCA with its anti-inflammatory properties may reduce inflammation and favor the production of pro-inflammatory cytokines, attenuating tissue inflammation^28^. BCA has also exerted vasodilatory effects, which may help improve blood flow and tissue perfusion during reperfusion following Ischemia. Enhanced vasodilation can deliver more oxygen and nutrient transport to the ischemic area, potentially reducing the extent of injury^29^.

This study showed that IR injury increased the MDA content and TOS value and lowered the TAS value. BCA treatment improved the scores because it has many biological activities (Table 1). BCA is a good free radical scavenger, and it induces the antioxidant system after IR, especially its antioxidant properties. IR causes the disruption of the cerebral cortex and degeneration of neurons. Pathological alterations are restored after BCA treatment (Figures 1A–C). Due to the neuroprotective effects of BCA, the cerebral cortex is histologically improved by BCA treatment after IR injury.

BBB protects the delicate nervous tissue from pathogens and microbes and its maintenance is quite vital for cerebral homeostasis. BBB endothelial cells help the regulation of BBB by induction of mechanical induction^30^. BECs are different from other peripheral endothelial cells such as possessing low adhesion molecules, high number of mitochondria, and high polarization^2^. Impairment of BBB causes alteration in the semi-selective permeability of BBB, leading to numerous neurological disorders. IR injury deteriorates the BBB and causes the upregulation of pro-inflammatory cytokines (e.g., TNF-α, IL-1β, and IL-6)^31^. BCA is a medicinal plant with many pharmacological activities such as anti-inflammatory and antioxidant properties. Guo et al., showed that BCA protected the neural tissue against the cerebral IR via oxidative stress and inflammation pathway^32^. El-Sayed et al.^33^ showed that BCA had neuroprotective effects in an epileptic animal model via modulation of inflammatory and autophagy pathways.

In this study, cerebral IR injury caused the disruption of BBB and reduced immunoreactivity of BBB. Administration of BCA upregulated the BBB expression and restored the BBB because its expression was increased compared with the IR group (Table 1, Figures 1D–F).

Although phytotherapy is acknowledged as a healing approach endorsed by national health authorities, it is still not officially recognized as a medical specialty^34^. However, we suggest that BCA treatment may modulate the components of BBB via induction of inflammation pathway and anti-oxidative stress mechanism.

## CONCLUSION

Cerebral IR injury causes the generation of free radicals and deteriorates the cerebral histology and BBB. With its antioxidant, BCA treatment reduced the ROS generated during IR injury and promoted the cellular scavenging system. Additionally, with its anti-inflammatory properties, BCA restored the BBB by modulating the inflammatory response pathway after cerebral IR injury.
